# Contributions of a survey and retrospective cohort study to the planning of a randomised controlled trial of corticosteroids in the treatment of paediatric septic shock

**DOI:** 10.1186/s13063-018-2664-x

**Published:** 2018-05-21

**Authors:** Anna Liu, Kusum Menon

**Affiliations:** 1 University of Ottawa, Ottawa, K1H 8M5 Canada; 2 Children’s Hospital of Eastern Ontario, 401 Smyth Road, Ottawa, ON K1H 8L1 Canada

**Keywords:** Shock, Paediatric, Intensive care units, Retrospective studies, Surveys and questionnaires, Randomised control trial

## Abstract

**Background:**

Randomised controlled trials (RCTs) are challenging to conduct in a paediatric critical-care environment. Background work, including surveys and observational studies, is often used to determine disease estimates, sample sizes and design protocols when planning such RCTs. Our objective was to determine the necessity of performing a survey or a retrospective chart review or both when planning an RCT on corticosteroids in the treatment of paediatric septic shock.

**Methods:**

We compared information on corticosteroid use for moderate to severe paediatric septic shock obtained from a survey of physician beliefs and stated practices with that obtained from a retrospective cohort study. The survey was conducted between February and March 2012 and the retrospective study included children from birth to 17 years of age admitted from January 2010 to June 2011. The survey and the retrospective study were conducted at four academic tertiary care centres in Canada.

**Results:**

Survey responses from 23 physicians and retrospective data from 81 septic shock patients were included. The survey identified time to discontinuation of vasoactive infusions as the most feasible and clinically important outcome for an RCT on corticosteroids for paediatric septic shock. The retrospective chart review provided means and standard deviations for the suggested primary outcome, from which we could estimate sample sizes and justify the minimal clinically important difference. The survey found that physicians believe that patients with severe septic shock were most likely to benefit from corticosteroid administration but the majority stated they would be unwilling to randomise such patients, suggesting a lack of individual physician equipoise. The combined information from the survey and retrospective study suggested that enrolment of patients with moderate septic shock would be more feasible but that strategies would still have to be implemented to prevent open-label corticosteroid use.

**Conclusions:**

The survey provided valuable information on the choice of primary outcome, target population and physician equipoise. The retrospective study provided estimates of patient numbers, the minimal clinically important difference, evidence for community equipoise and physician practice patterns. Strong consideration should be given to performing both types of studies prior to conducting RCTs in paediatric critical-care environments.

## Background

Randomised controlled trials (RCTs) are challenging to conduct in paediatric critical care yet they are crucial for guiding clinical practice. Barriers to conducting these RCTs include difficulties obtaining informed consent [1], resistance to randomisation [2] and limited funding opportunities [3]. Paediatric critical-care RCTs tend to be fewer in number, single-centred and have smaller sample sizes than adult RCTs [4]. Due to these challenges, careful background work is necessary to determine disease estimates, target populations, sample sizes and whether equipoise on the study question exists prior to conducting these trials. Surveys and retrospective chart reviews are often used for this background work [5, 6]. Surveys are inexpensive, easy to administer and may be completed in a timely manner. However, survey results may be difficult to interpret due to potential sampling bias, low response rates and response bias [7]. Retrospective cohort studies provide actual patient data, but usually require funding and are more labour intensive and subject to confounding and misclassification bias [6]. Although the relative merits of these two methodologies are well known, the utility of the specific information obtained using each of these methods in planning a prospective clinical trial has not been evaluated.

Corticosteroid use in the treatment of paediatric septic shock has been debated in the literature for over 40 years [8]. Despite numerous editorials [9], reviews [8, 10] and a limited number of small RCTs [11], the role of corticosteroids for treating paediatric septic shock remains unclear. Furthermore, despite this lack of evidence, it is unclear whether individual physicians have equipoise on corticosteroid use for paediatric septic shock [12]. As such, it is uncertain whether an RCT on the use corticosteroids in the treatment of paediatric septic shock would be feasible. Therefore, in preparation for a future trial, both a survey and retrospective cohort study were conducted to assess current beliefs and practices with regard to corticosteroid use for paediatric septic shock. Our objective was to compare the specific information obtained from each of these respective study designs to determine the need to conduct both types of studies as background work for future RCTs in paediatric critical care.

## Methods

We examined responses from questions of a previously published physician survey conducted between February and March 2012 [6] and data obtained from a previously published retrospective cohort study conducted between January 2010 and June 2011 [7]. The original survey included physicians from all academic centres across Canada. However, for this study, only survey responses obtained from paediatric intensive-care physicians at the four academic tertiary care centres that participated in the retrospective chart review were included. The survey included demographic questions, questions on physicians’ practice for patients with septic shock and questions regarding their beliefs relating to a RCT on the use of hydrocortisone versus placebo in the treatment of paediatric septic shock. Each non-demographic question within the survey included selections from choices and, where appropriate, an ‘other’ category where free-text comments could be inserted.

The original cohort study included data from 327 patients with moderate to severe shock from all causes. For this study, we extracted data from the septic shock sub-group, which included 81 patients from birth to 17 years of age with moderate to severe septic shock. The survey and retrospective study both defined moderate shock as hypotension despite a minimum of 60 cm^3^/kg of fluid and at least one vasoactive infusion. Retrospective data spanned a 1-year period for all but one centre, for which data was collected for 3 months due to its significantly larger patient population. Descriptive statistics are presented for the surveys and retrospective data. A comparison of proportions was performed using a chi-squared test or ANOVA for multiple comparisons between centres (SPSS version 24, IBM Corporation, New York).

## Results

**Fig. 1:**
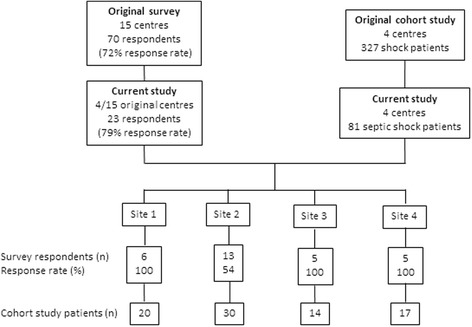
Flow diagram of survey respondents and cohort study patients for current study

The overall participant and patient flow diagram is shown in Fig. 1. All 23 physicians included in the survey were also responsible for patient care during the period of the retrospective cohort study.

### Eligible patient estimates

Less than half of surveyed physicians (11/23, 48%) correctly estimated the number of patients with moderate to severe shock treated at their site per year, with incorrect estimates being both higher and lower than the observed numbers. The retrospective cohort study, however, provided precise numbers of patients with mild, moderate and severe septic shock.

### Determination of the primary outcome for a trial

In total, 61% of respondents (14/23) stated that time to discontinuation of all vasoactive infusions was the most feasible and clinically important primary outcome for an RCT on the use corticosteroids in the treatment of paediatric septic shock. The retrospective chart review provided mean values with standard deviations for time on vasoactive infusions for those who did and did not receive hydrocortisone, from which we could estimate sample sizes for a future trial. None of the physicians surveyed chose mortality as the most feasible and clinically important primary outcome. In addition, the retrospective chart review found a baseline mortality rate of 11.7% for paediatric septic shock. As previously shown, this would require a sample size of 7682 patients to demonstrate an absolute mortality reduction of 2% (relative reduction of 17%) or 1764 patients for an absolute mortality reduction of 4% (relative reduction of 34%) [13]. Neither sample size is likely to be feasible given that the largest paediatric septic shock trial required pharmaceutical industry funding and 104 sites in 18 countries to recruit 477 patients in 3 years [14].

### Target population for corticosteroid administration in septic shock

**Fig. 2:**
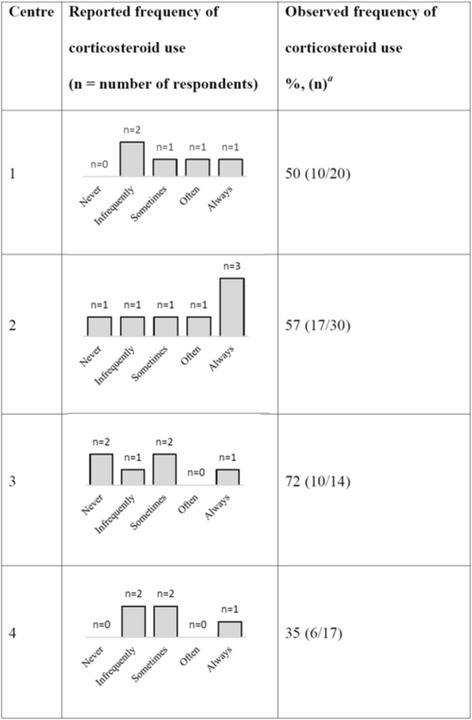
Comparison of reported versus observed use of corticosteroids in septic shock patients. ^*a*^The frequency of corticosteroid use is reported as the number of septic shock patients for whom corticosteroids were administered divided by the number of patients with septic shock

When physicians were asked how likely they were to administer corticosteroids for septic shock, their responses varied within and between each centre. Eight physicians stated that they often or always administered corticosteroids (35%, 8/23) and nine physicians stated that they infrequently or never did (9/23, 39%). The observed frequency of corticosteroid use for septic shock did not vary significantly between centres (36%, 6/17; 50%, 10/20; 57%, 17/30; 71%, 10/14; *P* = 1.00) and there was an overall observed rate of 53% (43/81). A comparison of stated and observed practices regarding corticosteroid administration in septic shock is shown in Fig. 2. Despite the low response rate from site 2, the diversity of responses is similar in all centres.

The percentage of physicians who stated they would administer corticosteroids, would be willing to randomise patients into an RCT and would administer open-label steroids at each of three levels of vasotropic support is shown in Table 1. The percentage of patients who received corticosteroids at each level of vasotropic support is also shown in Table 1. A statistically higher percentage of physicians stated they would administer corticosteroids to patients who were on two or more vasoactive drugs compared to those on one high dose or one low dose of a vasoactive drug (95.7%, 22/23 vs 52.2%,12/23 vs 8.7%, 2/23; *p* <  0.0001). However, this is also the population of physicians who were least likely to randomise patients in a trial of corticosteroids for paediatric septic shock (34.8%, 8/23 vs 69.6%, 16/23 vs 65.2%, 15/23; *p* <  0.0001) and were most likely to administer open-label steroids (87%, 20/23 vs 30.4%, 7/23 vs 4.3%, 1/23; *p* <  0.0001).

**Table 1:** Determination of target population for an RCT on the use of corticosteroids for paediatric septic shock

Population	60 cm^3^/kg of fluid + 1 low dose of vasoactive drug	60 cm^3^/kg of fluid + 1 high dose of vasoactive drug	60 cm^3^/kg of fluid + ≥2 types of vasoactive drug	*P* value
Physicians who administer corticosteroids % (*N*)	8.7 (2/23)	52.2 (12/23)	95.7 (22/23)	< 0.0001
Patients who received corticosteroids % (*N*)^a^	6.4 (5/81)	23.9 (19/81)	50.8 (41/81)	< 0.0001
Physicians who would randomize patients into an RCT % (*N*)	65.2 (15/23)	69.6 (16/23)	34.8 (8/23)	0.035
Physicians who would prescribe open-label steroids % (*N*)	4.3 (1/23)	30.4 (7/23)	87.0 (20/23)	< 0.0001

^a^Data from the retrospective cohort study. All other data were from the survey

*RCT* randomised controlled trial

Although 95.7% (22/23) of physicians stated they would administer corticosteroids to patients requiring two or more vasoactive medications, only 50.8% (41/81) of such patients received corticosteroids (*p* <  0.0001). These results were unchanged if the largest centre, which had the lowest survey response rate, was removed from the analysis (100%, 16/16; *p* = 1.0 and 47.1%, 24/51; *p* = 1.0).

### Existence of equipoise on corticosteroid administration for septic shock

A summary of stated and observed corticosteroid administration practices is shown in Table 1. Only 35% of physicians said they would be willing to randomise septic shock patients who had received 60 cm^3^/kg of fluid and were on at least two vasoactive infusions (the population of interest) into an RCT of corticosteroids versus placebo. In addition, 87% of respondents stated that they would administer open-label steroids to patients meeting these criteria if they were randomised. Interestingly however, only 50% of such patients actually received corticosteroids in the retrospective review. Of physicians, 70% stated they would be willing to randomise slightly less sick patients who had received 60 cm^3^/kg of fluid and were on one high dose of vasoactive infusion but 30% of those surveyed said they would still consider open-label corticosteroids for this group of randomised patients. In the retrospective study, 24% of such patients received corticosteroids. The combined information obtained from the survey and the retrospective study suggests that enrolment of slightly less sick patients may be feasible but that strategies will still have to be considered and implemented to prevent open-label corticosteroid use.

### Dose and duration of corticosteroid therapy

Altogether, 65% of survey respondents reported using the equivalent of 1 mg/kg/dose of hydrocortisone q6h and 26.1% used 2 mg/kg/dose q6h. In the retrospective study, 56.3% of patients received 1 mg/kg/dose q6h and 26.7% received 2 mg/kg/dose q6h providing strong evidence for consistency between beliefs and practices for hydrocortisone dosing for paediatric septic shock. There was significant variation in stated and observed practices within and between centres, suggesting that there is no standard duration of hydrocortisone therapy for paediatric septic shock. Therefore, the RCT protocol would require a physiologic rationale for duration of therapy rather than one based on an accepted standard of care.

### Feasibility of adrenal function testing of septic shock patients

In total, 78% of physicians (18/23) stated that they sometimes, often or always conducted adrenal axis testing prior to giving corticosteroids to patients in shock, but only 5% (4/78) of patients who received corticosteroids actually underwent testing. In each of the four centres, equal percentages of physicians reported using cortisol levels and adrenocorticotropic hormone (ACTH) testing for patients with shock. However, a larger proportion of patients who underwent adrenal function testing had cortisol levels measured compared to ACTH testing (73.3%, 11/15 versus 26.7%, 4/15; *P* = 0.012). The discrepancy between stated and observed rates of adrenal function testing prompted our team to explore potential barriers to adrenal testing rather than mandating its inclusion in our protocol.

### Information gained in the survey and cohort studies

A summary of the information gained from each of the respective study designs and its effect on the ultimate design of the future RCT are shown in Table 2.

**Table 2:** Summary of information gained from the survey and cohort study

Information needed	Information obtained from each study design		Information gained by doing both	Effect of information obtained on RCT protocol
	Survey study	Cohort study		
Overall frequency of steroid use for fluid resistant septic shock	Wide variation in individual physician responses within and between centres	Overall steroid use varied from 35.3% to 71.4% between centres	Despite survey responses to the contrary, one centre had a high rate of empiric corticosteroid administration	Centres with a high rate of steroid administration *and* centres with ≥50% of respondent physicians stating they often or always administer steroids for septic shock were not included in the RCT
Use of steroids in patients who received 60 cm^3^/kg of fluid and were on two or more vasoactive agents	95.6% of physicians surveyed stated they would administer steroids to such patients	50.8% of such patients actually received steroids	The discrepancy between the survey and cohort study findings suggests that physicians believe there may be benefit to corticosteroids but do not always administer them	This discrepancy provided support for the existence of community equipoise, which was needed as justification for the grant to fund the RCT
Performance of adrenal testing	The majority of physicians stated that they sometimes, often or always perform adrenal testing prior to steroid use	Only 5.1% of patients had adrenal testing performed prior to steroid use	The discrepancy between what physicians say they do versus what they did regarding adrenal testing suggests that physicians may believe that adrenal testing should be performed but rarely do	We identified barriers to conducting adrenal testing including delays in obtaining results, difficulty in interpreting results and the cost of conducting the test. Due to these findings, adrenal testing was not required as part of the protocol
Physician willingness to randomise patients	84.3% would be willing to randomise patients on one high dose of vasoactive medication. However, 74.3% would start open-label steroids in patients requiring two high doses of vasoactive medication	It was not possible to determine willingness to randomise but we observed a lower rate of steroid use (50.8%) in the patient group for which 74.3% of respondents said they would administer steroids	The lower rate of actual steroid administration suggests that physicians might be open to randomisation and protocol adherence in the target population but that open-label steroid use would be a significant threat to the feasibility of the study	This finding emphasised the need for a pilot RCT with close monitoring of both the frequency and reported reasons for open-label steroid use
Dose of hydrocortisone therapy	65.2% reported using the equivalent of 1 mg/kg/dose q6h and 26.1% used 2 mg/kg/dose q6h	56.3% of patients received 1 mg/kg/dose q6h and 26.7% received 2 mg/kg/dose q6h	The majority of physicians used 1 mg/kg/dose of hydrocortisone with 2 mg/kg/dose being a common second choice	The paediatric literature varies significantly regarding the dose of hydrocortisone for septic shock. However, given the consistency of the survey and cohort study data, we opted to use an initial bolus of 2 mg/kg/dose followed by 1 mg/kg/dose q6h

*RCT* randomised controlled trial

## Discussion

We used a specific research question to examine the information obtained from a survey and a retrospective cohort study to evaluate their respective utility in designing a prospective RCT. We observed that both types of study were needed to determine the numbers of eligible patients, justify the primary outcome measure, calculate the sample size, select the target population and establish the feasibility of the selected protocol.

Estimating numbers of potentially eligible patients is one of the most important tasks when planning an RCT. Due to the limited literature on paediatric critical care [3], there is heavy reliance on surveys when deriving estimates [15–18]. We found that the majority of physicians incorrectly estimated the number of patients admitted with shock at their centre, suggesting that reliance on survey data alone may not provide accurate estimates of eligible patients and that despite the additional time and costs involved, observational studies are needed [16]. In addition, the inaccuracy of the patient estimates obtained from the survey suggest that it may be wise to omit such questions from future surveys to shorten the surveys and limit survey fatigue [19].

Choosing an appropriate primary outcome for an RCT is another important component of RCT study design and is crucial for sample size calculations [20], determination of feasibility and interpretation of the relevance of a trial [21]. The choice of primary outcome is often justified based on the available literature. However, this approach may not always provide the most appropriate primary outcome. A systematic review of previous paediatric septic shock trials found that mortality was the most commonly used primary outcome [13] but its use may be limited by low baseline mortality rates in developed countries [22]. Our survey also supported the limited utility of mortality as a primary outcome in paediatric septic shock trials and provided a formalised method for stakeholders to suggest alternative clinically important outcomes. The survey results suggested time to haemodynamic stability as the most clinically important and feasible primary outcome and the retrospective cohort study allowed us to determine means and standard deviations for this outcome measure. Methodologists have highlighted the need for rigour when determining the minimal clinically important difference [23] and our retrospective study provided an estimate of the difference in time to haemodynamic stability between those who did and did not receive hydrocortisone. Thus, both study designs contributed to the justification of haemodynamic stability as a possible primary outcome. Interestingly, none of the respondents from our survey suggested a patient-centred measure as the most important outcome for an RCT. However, a more recent survey found quality of life to be the most commonly chosen primary outcome for a future RCT on using steroids in the treatment of paediatric septic shock [24], which may reflect a growing focus of granting agencies on patient-centred outcomes.

The next step in designing an RCT is to determine the target population. Inclusion criteria are often chosen using a balance between scientific rationale and feasibility and researchers have often used surveys to establish clinical criteria for RCTs in paediatric critical care [15, 17, 18]. Physiologic evidence suggests there is a potential benefit of prescribing corticosteroids to haemodynamically unstable patients [25–28], whom our survey respondents defined as those who had received 60 cm^3^/kg of fluid and were on two or more vasoactive infusions. Our survey showed that only a third of physicians would be willing to randomise such patients into an RCT and that 87% of physicians would administer open-label corticosteroids to such patients if randomised. This suggests a lack of equipoise amongst individual physicians, which could pose a significant threat to the feasibility of a trial if not specifically addressed. Interestingly though, the cohort study showed that almost exactly half of such patients actually received corticosteroids, suggesting that there may, in fact, be community equipoise on the use of corticosteroids for paediatric septic shock. The lack of clear evidence for benefit or harm from corticosteroids in treating septic shock and the presence of community equipoise together provide the ethical mandate for recruitment into an RCT, further supporting the additional value of the cohort study in preparing for this RCT.

The last step in the design of an RCT is finalising the details of the actual protocol. The survey and cohort study were used to explore the duration of hydrocortisone therapy to be used in the protocol. However, the significant variation in both stated and actual practice showed that there is no accepted standard of care, which provides good justification for the use of limited physiologic data and adult studies to determine the duration of hydrocortisone for the RCT. Finally, the majority of physicians surveyed stated that they would conduct adrenal axis testing prior to corticosteroid administration, but the retrospective data revealed that such testing was rarely performed. The survey results suggested that physicians are aware of the recommendations for adrenal axis testing [29], but the cohort study found that these recommendations were not followed. This discrepancy between stated and observed practices led us to explore potential barriers to adrenal axis testing. These included the cost of ACTH, the lack of real-time availability of test results [30] and difficulty in interpreting the results [31]. Based on these findings, adrenal testing was not included in the protocol.

Finally, it is important to acknowledge the potential limitations of surveys and retrospective cohort studies. Surveys are subject to sampling and response bias, which may lead to a misinterpretation of true beliefs [32]. In addition, concerns have been raised about survey fatigue, which may limit response rates and the quality of information obtained [6]. However, surveys are inexpensive, easy to administer and most importantly, still provide a formalised structure by which to obtain expert opinions. In our specific example, the survey provided valuable information on choice of primary outcome, inclusion criteria, existence of equipoise and protocol details. Retrospective studies may be subject to misclassification bias and incomplete data but provide an important source of preliminary estimates [19] and have played an important role in the design of adult critical-care trials [33]. Our chart review provided us with estimates of eligible patients, justification for the minimal clinically important difference and actual practice patterns.

The strengths of this study include the inclusion of multiple centres and the novel comparison of the contributions of information obtained from a survey versus a retrospective study in the planning of a paediatric critical-care RCT. There are, however, several limitations to this study. Survey non-respondents may have cared for patients within the retrospective study, thus affecting observed values but not influencing reported practices. However, this limitation applies only to one site as the other sites had 100% survey response rates. Lastly, we compared only one survey and only one retrospective study of a single topic in a specialised group of physicians and therefore, our observations may not be applicable to other studies or settings.

## Conclusions

The survey provided valuable information on physician preferences for the choice of primary outcome, identification of the target population and individual physician equipoise. The retrospective cohort study provided estimates of eligible patient numbers, the minimal clinically important difference, evidence of community equipoise and actual physician practice patterns. A comparison of the information obtained from each of the two study methods provided necessary insights into the feasibility of recruiting our target population and of conducting adrenal function testing. Strong consideration should be given to performing both types of study prior to conducting an RCT in a paediatric critical-care environment.

## Abbreviations

ACTH: Adrenocorticotropic hormone
RCT: Randomised controlled trial

## References

[1] Menon K, Ward R. A study of consent for participation in a non-therapeutic study in the pediatric intensive care population. J Med Ethics. 2014;40:123–6 10.1136/medethics-2012-101075 23345569

[2] Hamm MP, Scott SD, Klassen TP, Moher D, Hartling L (2012). Do health care institutions value research? A mixed methods study of barriers and facilitators to methodological rigor in pediatric randomized trials. BMC Med Res Methodol.

[3] Duffett M, Choong K, Hartling L, Menon K, Thabane L, Cook DJ (2013). Randomized controlled trials in pediatric critical care: a scoping review. Crit Care.

[4] Duffett M, Choong K, Hartling L, Menon K, Thabane L, Cook DJ (2015). Pilot Randomized Trials in Pediatric Critical Care: A Systematic Review. Pediatr Crit Care Med.

[5] Hulley S, Cummings S, Browner W, Grady DG, Newman TB (2013). Designing Clinical Research.

[6] Vassar M, Holzmann M (2013). The retrospective chart review: important methodological considerations. J Educ Eval Health Prof.

[7] Bennett C, Khangura S, Brehaut JC, Graham ID, Moher D, Potter BK, Grimshaw JM (2010). Reporting guidelines for survey research: an analysis of published guidance and reporting practices. PLoS Med.

[8] Menon K, Wong HR (2015). Corticosteroids in Pediatric Shock: A Call to Arms. Pediatr Crit Care Med.

[9] Zimmerman JJ (2013). Expanding the conversation regarding adjunctive corticosteroid therapy for pediatric septic shock*. Pediatr Crit Care Med.

[10] Annane D, Bellissant E, Bollaert PE, Briegel J, Keh D, Kupfer Y (2004). Corticosteroids for severe sepsis and septic shock: a systematic review and meta-analysis. BMJ.

[11] Menon K, McNally D, Choong K, Sampson M (2013). A systematic review and meta-analysis on the effect of steroids in pediatric shock. Pediatr Crit Care Med.

[12] Menon K, McNally D, O’Hearn K, Acharya A, Wong HR, Lawson M, Ramsay T, McIntyre L, Gilfoyle E, Tucci M, Wensley D, Gottesman R, Morrison G, Choong K. A randomized Controlled Trial of Corticosteroids in Pediatric Septic Shock: A Pilot Feasibility Study. Pediatr Crit Care Med. 2017;18:505–12 10.1097/PCC.0000000000001121 PMC5457353 28406862

[13] Menon K, McNally JD, Zimmerman JJ, Agus MS, O’Hearn K, Watson RS, Wong HR, Duffett M, Wypij D, Choong K (2017). Primary Outcome Measures in Pediatric Septic Shock Trials: A Systematic Review. Pediatr Crit Care Med.

[14] Nadel S, Goldstein B, Williams MD, Dalton H, Peters M, Macias WL, Abd-Allah SA, Levy H, Angle R, Wang D, Sundin DP, Giroir B (2007). Drotrecogin alfa (activated) in children with severe sepsis: a multicentre phase III randomised controlled trial. Lancet.

[15] Mannarino CN, Faustino EV (2016). Clinical equipoise on prophylaxis against catheter-associated thrombosis in critically ill children. J Crit Care.

[16] Menon K, McNally JD, Choong K, Ward RE, Lawson ML, Ramsay T, Wong HR (2013). A survey of stated physician practices and beliefs on the use of steroids in pediatric fluid and/or vasoactive infusion-dependent shock. Pediatr Crit Care Med.

[17] Hirshberg EL, Sward KA, Faustino EV, Nadkarni VM, Agus MS, Morris AH, Lacroix J (2013). Clinical equipoise regarding glycemic control: a survey of pediatric intensivist perceptions. Pediatr Crit Care Med.

[18] Scholefield BR, Duncan HP, Morris KP (2010). Survey of the use of therapeutic hypothermia post cardiac arrest. Arch Dis Child.

[19] O’Reilly-Shah VN (2017). Factors influencing healthcare provider respondent fatigue answering a globally administered in-app survey. PeerJ.

[20] Menon K, McNally JD, Choong K, Lawson ML, Ramsay T, Hutchison JS, Foster J, Wong HR (2015). A cohort Study of Pediatric Shock: Frequency of Corticosteriod Use and Association with Clinical Outcomes. Shock.

[21] Pocock SJ, Stone GW (2016). The Primary Outcome Is Positive - Is That Good Enough?. N Engl J Med.

[22] Kissoon N, Carcillo JA, Espinosa V, Argent A, Devictor D, Madden M, Singhi S, van der Voort E, Latour J (2011). World Federation of Pediatric Intensive Care and Critical Care Societies: Global Sepsis Initiative. Pediatr Crit Care Med.

[23] Cook JA, Hislop J, Altman DG, Fayers P, Briggs AH, Ramsay CR, Norrie JD, Harvey IM, Buckley B, Fergusson D, Ford I, Vale LD (2015). Specifying the target difference in the primary outcome for a randomised controlled trial: guidance for researchers. Trials.

[24] Merritt C, Menon K, Agus MSD, Choong K, McNally D, O’Hearn K, Watson RS, Wong HR, Duffett M, Wypij D, Zimmerman JJ (2018). Beyond Survival: Pediatric Critical Care Interventional Trial Outcome Measure Preferences of Families and Healthcare Professionals. Pediatr Crit Care Med.

[25] Wehling M (1997). Specific, nongenomic actions of steroid hormones. Annu Rev Physiol.

[26] Munck A, Mendel DB, Smith LI, Orti E (1990). Glucocorticoid receptors and actions. Am Rev Respir Dis.

[27] Sasidharan P (1998). Role of corticosteroids in neonatal blood pressure homeostasis. Clin Perinatol.

[28] Shi WL, Zhang T, Zhou JR, Huang YH, Jiang CL (2016). Rapid permissive action of dexamethasone on the regulation of blood pressure in a rat model of septic shock. Biomed Pharmacother.

[29] Davis AL, Carcillo JA, Aneja RK, Deymann AJ, Lin JC, Nguyen TC, Okhuysen-Cawley RS, Relvas MS, Rozenfeld RA, Skippen PW, Stojadinovic BJ, Williams EA, Yeh TS, Balamuth F, Brierley J, de Caen AR, Cheifetz IM, Choong K, Jr CE, Cornell T, Doctor A, Dugas MA, Feldman JD, Fitzgerald JC, Flori HR, Fortenberry JD, Graciano AL, Greenwald BM, Hall MW, Han YY, Hernan LJ, Irazuzta JE, Iselin E, van der Jagt EW, Jeffries HE, Kache S, Katyal C, Kissoon NT, Kon AA, Kutko MC, MacLaren G, Maul T, Mehta R, Odetola F, Parbuoni K, Paul R, Peters MJ, Ranjit S, Reuter-Rice KE, Schnitzler EJ, Scott HF, Torres A, Weingarten-Abrams J, Weiss SL, Zimmerman JJ, Zuckerberg AL (2017). American College of Critical Care Medicine Clinical Practice Parameters for Hemodynamic Support of Pediatric and Neonatal Septic Shock. Crit Care Med.

[30] Manglik S, Flores E, Lubarsky L, Fernandez F, Chhibber VL, Tayek JA (2003). Glucocorticoid insufficiency in patients who present to the hospital with severe sepsis: a prospective clinical trial. Crit Care Med.

[31] Menon K, Ward RE, Lawson ML, Gaboury I, Hutchison JS, Hebert PC (2010). A prospective multicenter study of adrenal function in critically ill children. Am J Respir Crit Care Med.

[32] Duffett M, Burns KE, Adhikari NK, Arnold DM, Lauzier F, Kho ME, Meade MO, Hayani O, Koo K, Choong K, Lamontagne F, Zhou Q, Cook DJ (2012). Quality of reporting of surveys in critical care journals: a methodologic review. Crit Care Med.

[33] Cook D, Heyland D, Marshall J (2001). On the need for observational studies to design and interpret randomized trials in ICU patients: a case study in stress ulcer prophylaxis. Intensive Care Med.

